# White-nose syndrome in bats: illuminating the darkness

**DOI:** 10.1186/1741-7007-11-47

**Published:** 2013-04-15

**Authors:** Paul M Cryan, Carol Uphoff Meteyer, Justin G Boyles, David S Blehert

**Affiliations:** 1 US Geological Survey, Fort Collins Science Center, Fort Collins, CO 80526, USA; 2 US Geological Survey, National Center, 12201 Sunrise Valley Drive, MS 913, Reston, VA 20192, USA; 3 Southern Illinois University, Cooperative Wildlife Research Laboratory, Department of Zoology, Carbondale, IL 62901; 4 US Geological Survey, National Wildlife Health Center, Madison, WI 53711, USA

## 

Happy ten-year anniversary to *BMC Biology*! We can attest to the effectiveness of the journal in reaching a great diversity of scientists based on reader responses to our commentary [1] about bat white-nose syndrome (WNS) two years ago. WNS is still on course to rank among the most destructive wildlife diseases to emerge in recent history, and it has continued to have unprecedented effects on populations of hibernating bats in eastern North America. At the time of our last writing in November 2010, the cold-adapted fungus then presumed to cause WNS (*Geomyces destructans*) had spread about 1,300 km from an index site in New York (Figure 1). In those early years of the epizootic, WNS caused a staggering wave of mass mortality among all six species of hibernating bats that occur in north-eastern North America. Since November 2010, WNS has spread into eight additional US states and two more Canadian provinces (Figure 1), and has continued to cause mortality in those six species most affected during the early years of the epizootic. Although part of a mostly tragic story has continued to unfold as new areas are affected, anecdotal signs are emerging that all may not be lost when it comes to hibernating bats and WNS. Amid the continued large-scale population declines of certain species, we have yet to see mass mortality in some of the more westerly areas where the fungus was detected two winters ago (Figure 1). Also, recently disease without obvious mortality was diagnosed in gray bats (*Myotis grisescens*) - an endangered species thought by many two years ago to be at high risk of extinction from WNS. Clearly, large gaps in our understanding of WNS remain, but some have been filled since we last communicated with readers of *BMC Biology*.

**Figure 1:**
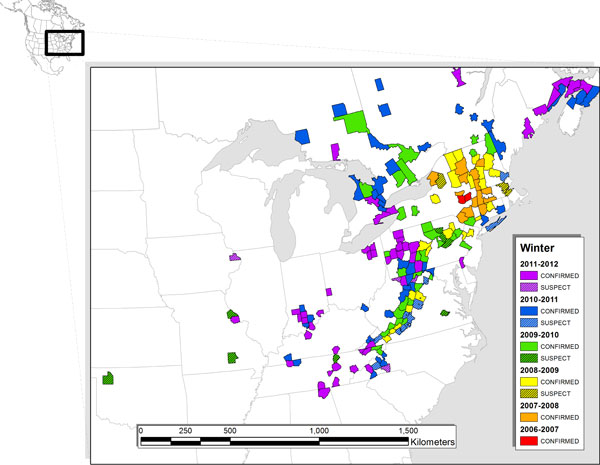
**The spread of bat white-nose syndrome and the fungus (*Geomyces destructans*) that causes the disease**. Since the winter of 2006-2007 (red), the disease (solid colors) as determined by histopathology analysis, and DNA of the fungal pathogen (striped colors) as identified by PCR analysis, have been detected across large expanses of eastern North America. Mass mortality has not yet been observed in the westernmost outlying area where presence of fungal DNA on a hibernating bat was first indicated by PCR during the winter of 2009-2010 (green striped area to far left).

A question two years ago was how a cold-adapted and slow-growing fungus could kill so many hibernating bats. At the time, most hypotheses tended to focus on energetic explanations in which bats infected with *G. destructans *prematurely run out of fat stores during hibernation. In our 2010 commentary we emphasized there was more to WNS than meets the naked eye and that fungal damage to wings during hibernation was far more severe and physiologically disruptive than appreciated. Since then, new discoveries have shed light on the unfortunate circumstances under which bats and *G. destructans *meet during winter. The emerging picture reveals potentially close linkages between physical damage caused by the fungus and the time and energy (stored fat) available to bats for coping with infection.

It is now very clear the fungus, *G. destructans*, is the cause of WNS in North America, but it has different effects on bats in Europe. Infection studies completed in two different laboratories with little brown bats (*Myotis lucifugus*) confirmed that the fungus causes the skin lesions characteristic of the disease [2] and death after approximately two to three months of hibernation [3]. A European isolate of the fungus also killed experimentally infected North American little brown bats [3]. However, although wild hibernating bats of Europe develop fungal skin lesions consistent with those seen in their North American counterparts [4], *G. destructans *does not appear to cause mass mortality of wild European bats. Current thinking is that *G. destructans *was accidentally translocated from Europe into a naïve assemblage of bat hosts through an index site in New York. Genetic studies comparing isolates of the fungus from North America and Europe are on-going, and the geographic source of this likely introduction remains a mystery. Together, these findings suggest that intercontinental differences in mortality may have more to do with susceptibility of resident bats or their infested habitats than differences in the pathogen itself.

From the earliest days of the investigation, fungal-influenced, hyper-frequent arousals from hibernation were hypothesized to cause premature fat depletion and death in WNS bats. Experimental evidence from laboratory infection trials, as well as observations from wild bats, recently confirmed that bats with WNS indeed arouse from torpor more frequently than uninfected bats, particularly during the later stages of disease severity [3,5]. Energy depletion due to fungal-altered arousals undoubtedly contributes to mortality. However, we still do not know what triggers these arousals. In 2010, we hypothesized that dehydration from infection might influence arousals and mortality [1]. There is new evidence of hypotonic dehydration and associated electrolyte depletion in WNS bats [6], as well as modeling that indicates the little brown bat, a species showing high susceptibility to WNS in North America, has greater rates of evaporative water loss than a similar-sized hibernating congener in Europe [7]. Although experimental evidence of dehydration (or other physiological processes) causing increased arousals is still lacking, dehydration remains a plausible explanation for both WNS arousal patterns and species differences in susceptibility to WNS.

The anecdotal differences in susceptibility to WNS among bats in North America that we noted in 2010 have been substantiated by analysis of historical census data [8]. As more information is synthesized from outlying regions, including Europe, the question remains - what influences differences in susceptibility and disease severity among different species of bats? We now know that the underground habitats relied on by hibernating bats for winter survival can serve as long-term reservoirs of the fungal pathogen [9]. This year we also saw new information indicating that isolates of *G. destructans *from North America and Europe all show maximum rates of growth in culture from approximately 12 to 16°C, although variation in growth rates among geographically distinct isolates cultured at the same temperatures in lab settings remain unexplained [10]. We previously emphasized a possible connection between humidity inside a hibernation site and susceptibility to WNS, which, along with evidence of more rapid population declines in warmer hibernacula, has now been demonstrated with field data [8]. Germination of conidial fungi is often dependent on surface moisture, and considering the patterns described above with WNS and *G. destructans *in culture, it may be that fungal virulence is governed in large part by humidity and temperature conditions on skin surfaces of hibernating bats. Different bat species rely on different humidity and temperature conditions in their underground winter habitats. It follows that variations in humidity and temperature within and among hibernation sites could have major influences on the extent and severity of infection by *G. destructans *among bat species, regions, and continents.

One of the more encouraging findings since we last wrote is that bats can recover from WNS infection, but recovery is a physiologically challenging process that requires time and energy [11,12]. Following emergence from winter hibernation, both captive and wild bats have been documented to overcome infection, and wing healing is complete after several weeks (Figure 2). The immune response and healing process likely requires consistent euthermic (approximately 38°C) body temperatures, yet hibernating bats do not store sufficient fat to remain euthermic for such long periods. During winter, inflammatory cell response at the site of infection by *G. destructans *is mostly delayed until arousal from hibernation in spring and new evidence suggests that, if the fungal infection of skin has been extensive during hibernation, this inflammatory response has the potential to fatally overwhelm the host instead of contributing to healing and recovery [13]. We hypothesized previously that physiological damage to wings caused by the fungus during winter could cause mortality, but it now appears that clearing the infection after arousal could contribute to additional life-threatening physical damage to wings [13]. Another new study related to survival of bats with WNS suggested a possible connection between environmental conditions outside of bat hibernacula and WNS mortality, with a higher likelihood of mortality occurring at hibernation sites situated in cold, seasonally variable, high-elevation regions [14]. Conditions outside of hibernacula may influence disease outcome for bats that transiently emerge from hibernation during winter. Bats that hibernate in areas with shorter winters or where mid-winter feeding opportunities allow them to supplement energy reserves for an earlier euthermic immune response seem less likely to be overwhelmed by infection with *G. destructans*. Together, these new discoveries highlight that WNS disease processes may vary across many scales, of which hibernation environments and the physiological status and behavioral characteristics of the hosts are but a few.

**Figure 2:**
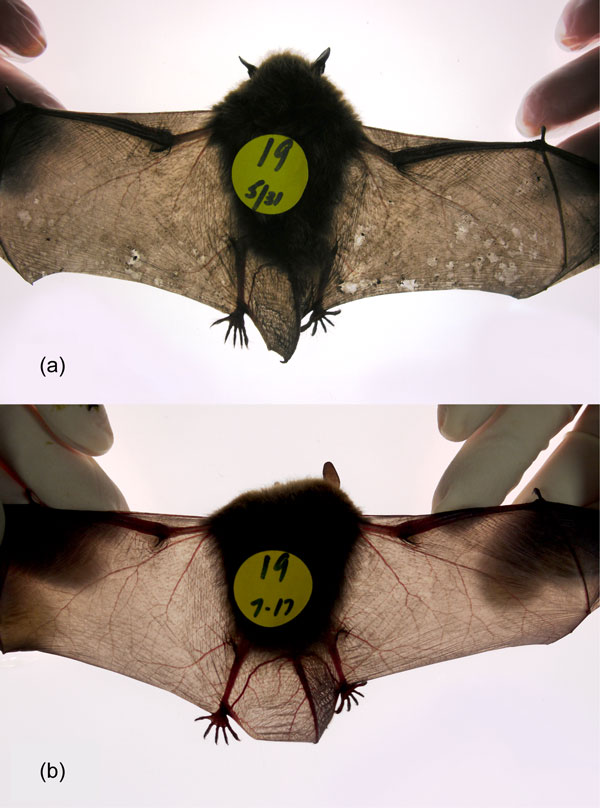
**Recent studies show that bats can recover from white-nose syndrome**. Sequential back-lit photographs showing wing healing over time in a little brown bat (*Myotis lucifugus*) that was naturally infected by the fungus *Geomyces destructans *during hibernation and then brought into captivity for supportive care, as reported by Meteyer *et al. *2011 [11]. This type of healing was observed in many of the 25 bats that recovered from white-nose syndrome by the end of that 70-day study; 31 May **(a) **the wings show areas of increased density and contraction due to fungal damage, as well as translucent areas indicative of thinning or loss of epidermis associated with immune response and healing. On 17 July **(b)**, the same wings show recovery without evidence of lesions or scarring. Images modified from Meteyer *et al. *2011 [11], page 622.

We are grateful to the Editors of *BMC Biology *for publishing what was, at the time, a rather speculative commentary, and for providing us another opportunity to update journal readership about a situation that has continued to unfold. We now understand the pathogenesis of WNS better than we did when our commentary was originally published; however, wide gaps remain in our understanding of this important disease. The pathogenesis of WNS seems to involve a cascade of events across multiple host systems and processes, all occurring against a backdrop of high environmental variability and host diversity. Further advances in characterizing the most relevant aspects of the ecology and pathogenesis of this disease may require more-integrated and collaborative efforts. Yet, as we rapidly move forward toward answers to WNS, it will be more important than ever to ensure that we carefully weigh potential scientific advances against research use and disturbance of live bats, as their populations in North America face uncertain futures. Looking ahead, we strongly believe that continued research into the pathogenesis of WNS will be a vital part of responding to this unprecedented epizootic.

## Note

This article is part of the *BMC Biology *tenth anniversary series. Other articles in this series can be found at http://www.biomedcentral.com/bmcbiol/series/tenthanniversary.
